# Autoimmune polyglandular syndrome type 4: experience from a single reference center

**DOI:** 10.3389/fendo.2023.1236878

**Published:** 2023-10-23

**Authors:** Elisa Gatta, Valentina Anelli, Elena Cimino, Elena Di Lodovico, Elda Piovani, Irene Zammarchi, Giorgia Gozzoli, Virginia Maltese, Maria Cavadini, Barbara Agosti, Andrea Delbarba, Ilenia Pirola, Angela Girelli, Caterina Buoso, Francesca Bambini, Daniele Alfieri, Walter Bremi, Paolo Facondo, Roberto Lupo, Francesco Bezzi, Micaela Fredi, Anna Maria Mazzola, Elena Gandossi, Maura Saullo, Fiorella Marini, Massimo Licini, Letizia Chiara Pezzaioli, Laura Pini, Franco Franceschini, Chiara Ricci, Carlo Cappelli

**Affiliations:** ^1^ Department of Clinical and Experimental Sciences, SSD Endocrinologia, University of Brescia, ASST Spedali Civili of Brescia, Brescia, Italy; ^2^ UOC Medicina Generale ad indirizzo Metabolico e Diabetologico, ASST Spedali Civili of Brescia, Brescia, Italy; ^3^ Sindacato Unico Medicina Ambulatoriale Italiana e Professionalità dell’Area Sanitaria – SUMAI, Brescia, Italy; ^4^ Department of Clinical and Experimental Sciences, Rheumatology and Clinical Immunology, University of Brescia, ASST Spedali Civili of Brescia, Brescia, Italy; ^5^ Department of Clinical and Experimental Sciences, Gastroenterology Unit, University of Brescia, ASST Spedali Civili of Brescia, Brescia, Italy; ^6^ Department of Clinical and Experimental Sciences, Respiratory Medicine Unit, University of Brescia, ASST Spedali Civili of Brescia, Brescia, Italy

**Keywords:** autoimmune polyglandular syndrome, type I diabetes mellitus, autoimmune diseases, polyendocrinopathies, autoimmunity

## Abstract

**Purpose:**

To characterize patients with APS type 4 among those affected by APS diagnosed and monitored at our local Reference Center for Autoimmune Polyglandular Syndromes.

**Methods:**

Monocentric observational retrospective study enrolling patients affected by APS diagnosed and monitored in a Reference Center. Clinical records were retrieved and analyzed.

**Results:**

111 subjects (51 males) were affected by APS type 4, mean age at the onset was 23.1 ± 15.1 years. In 15 patients the diagnosis of APS was performed during the first clinical evaluation, in the other 96 after a latency of 11 years (range 1-46). The most frequent diseases were type I diabetes mellitus and celiac disease, equally distributed among sexes.

**Conclusions:**

The prevalence of APS type 4 is 9:100,000 people. Type I diabetes mellitus was the leading indicator of APS type 4 in 78% subjects and in 9% permitted the diagnosis occurring as second manifestation of the syndrome. Our data, showing that 50% of patients developed APS type 4 within the first ten years, don’t suggest any particular follow-up time and, more importantly, don’t specify any particular disease. It is important to emphasize that 5% of women developed premature ovarian failure.

## 1 Introduction

Autoimmune polyglandular syndromes (APS) are rare and registered orphan diseases (ORPHAcode ORPHA:282196) characterized by insidious presentation and circulating autoantibodies and lymphocytic infiltration of one or more endocrine glands, with possible additional involvement of non-endocrine organs, eventually leading to organ failure (1–3).

There is a broad heterogeneity of APS and these manifest sequentially with a variable time interval between the occurrence of the diseases (4). The original classification into 4 types by Neufeld et al. in 1980 was revised by Betterle and Zanchetta in 2003, sub-classifying APS type 3 into four different sub-groups (5, 6). Indeed, some authors considered only APS-1 and APS-2 and did not consider APS-3 and APS-4 as independent entities (2, 7, 8).

APS type 1 (ORPHA:3453), due to different mutations of the autoimmune regulator (AIRE) gene on chromosome 21, is characterized by the presence of chronic candidiasis, chronic hypoparathyroidism, and Addison’s disease (1, 6). It has onset in childhood with an estimated prevalence of 1:80,000 live births (9) and a slight female predominance (10).

APS type 2 (ORPHA:3143), associated with a genetic pattern of human leukocyte antigen (HLA) DR3/DR4, is characterized by the presence of Addison’s disease and autoimmune thyroid diseases and/or type 1 diabetes mellitus (T1DM) (1, 6). The onset is predominantly in young adulthood (2), with a prevalence of 1:20,000 and a sex ratio male/female 1:3 (11).

Autoimmune thyroid diseases associated with other autoimmune diseases (excluding Addison’s disease and/or hypoparathyroidism) fall under APS type 3 (ORPHA:227982) (1, 5, 12). This syndrome is subdivided into type 3A if associated with other endocrine diseases, type 3B if associated with gastrointestinal diseases, type 3C with skin, haemopoietic system or nervous system diseases and type 3D with rheumatic diseases (6). The actual incidence is estimated at 1:20,000 and it is three times more frequent among women (11, 13). APS type 4 (ORPHA:227990) includes all the different clinical combinations of autoimmune diseases not included in the previous groups and affecting an endocrine organ (with the exception of Addison’s disease, thyroid diseases, or hypoparathyroidism) in combination with at least one more endocrine or non-endocrine organs (1, 6). To the best of our knowledge, there is scarce clinical data and no epidemiological data on this category globally.

The aim of the present study was to characterize patients with APS type 4 among those affected by APS diagnosed and monitored at our local Reference Center for Autoimmune Polyglandular Syndromes.

## 2 Methods

### 2.1 Subjects

All the medical records of patients referred for autoimmune disease to the Units of Endocrinology, Diabetology, Gastroenterology, Rheumatology and Clinical Immunology at the ASST Spedali Civili in Brescia were retrospectively reviewed from January 2000 up to 30 November 2022. All the patients were screened for the most frequent autoimmune endocrinopathies annually, as well as for Addison’s disease and all autoimmune pathologies when clinically suspected. All patients affected by Autoimmune Polyglandular Syndrome, according to the ORPHAcode (ORPHA 282196, 3453, 3143, 227982, 227990), were included in this study. The study (ASST_BS_CLIN_PZ_SPA-BS) was approved by the local Ethics Committee (no 5517).

### 2.2 Clinical data collection

Clinical manifestations of APS type 4 [including type 1 diabetes mellitus or latent autoimmune diabetes in adults (LADA), premature ovarian failure, celiac disease, atrophic gastritis, inflammatory bowel disease, rheumatoid arthritis, systemic lupus erythematosus, scleroderma, Sjogren syndrome, mixed connective tissue disease, vasculitis, antiphospholipid syndrome, primary biliary cirrhosis, autoimmune hepatitis, alopecia areata, autoimmune urticaria, myasthenia gravis, multiple sclerosis, pernicious anemia, immune thrombocytopenia, vitiligo, seronegative arthritis, ankylosing spondylitis, psoriasis, pemphigoid], as well as related patient information such as sex, onset age or age at diagnosis of the first and subsequent APS manifestations, were retrospectively retrieved from medical records. Diagnosis was performed in accordance with good clinical practice by antibody serology tests and, where required, histopathological analysis (i.e., celiac disease, atrophic gastritis, systemic lupus erythematosus, scleroderma, vasculitis, autoimmune hepatitis). In agreement with ORPHANET classification all the patients affected or showing antibodies suggesting Addison’s disease, thyroid diseases, or hypoparathyroidism were excluded from this study.

### 2.3 Statistical analysis

All data were collected in an electronic case report database. Normal distribution was checked using the Shapiro-Wilk test. Latency results were nonnormally distributed and were not normalized by the usual procedures of data transformation; in these cases, the results are presented as a median, with minimum and maximum values. Comparison between groups and differences in proportion were calculated using the χ^2^ test for categorical variables and ANOVA for quantitative variables, as appropriate. Between-group comparison was performed using the Student’s T-test for unpaired data or Kruskal-Wallis test, as appropriate. The Kaplan-Meier curve was fitted to determine the APS type 4 diagnosis time. A *p-*value < 0.05 was considered statistically significant. The statistical analyses were performed using SPSS 20.0 software (SPSS, Inc., Evanston, IL, USA). The results are reported in compliance with the STROBE reporting guidelines for cross-sectional studies; the checklist is reported in **Supplementary File 1**.

## 3 Results

A total of 9164 patients were referred to the Units of Endocrinology, Diabetology, Gastroenterology, Rheumatology and Clinical Immunology for autoimmune diseases. Among these, 1161 (12.7%) were diagnosed with any autoimmune polyglandular syndromes in accordance with good clinical practice by antibody serology tests and, where required, histopathological analysis, such as for all patients with positive antibodies for celiac disease, atrophic gastritis and/or vasculitis. Among the 1161 patients with APS, 111 (9.6%) subjects (51 males) were affected by APS type 4 (47.8 ± 17.1 years old, range 20-85) and were enrolled in the present study.

The mean age at the onset of APS was 23.1 ± 15.1 years, with no significant difference between sexes (22.3 ± 15.2 vs. 23.8 ± 15.0 yrs, M/F, p = .611). APS type 4 was diagnosed during first clinical evaluation in 15/111 (13.5%) patients (Group 1): celiac disease and multiple sclerosis were concomitantly diagnosed during T1DM evaluation in 13 and 2 subjects, respectively (**Table 1**). These patients did not develop further diseases during follow-up (14.3 ± 8.6, range 1-33 yrs).

**Table 1:** Demographic and clinical characteristics of patients with diagnosis of APS type 4 at the first clinical evaluation.

Disease	Number of patients (%)	Sex M/F	Overall mean age of diagnosis (years)	Latency before second manifestation (years)	Total follow-up from first manifestation (years)
Type I diabetes mellitus and celiac disease	13 (86.7%)	8/5	23.5 ± 14.3	NA	13.7 ± 6.9
Type I diabetes mellitus and multiple sclerosis	2 (13.3%)	0/2	23.5 ± 4.9	NA	18.5 ± 20.5

NA, not applicable.

APS type 4 was diagnosed in 96 patients in the years following the first disease (range 1-46 yrs) (Group 2); in detail, the most frequent first clinical manifestations were T1DM in 72/96 (75%) patients, celiac disease in 9 (9%) and vitiligo in 4 (4%) (**Table 2**). Groups 1 and 2 were superimposable for age of disease onset (23.5 ± 13.3 vs. 23.0 ± 15.4 yrs, p=.292) and sex (8/7 vs 43/53, M/F, p=.537).

**Table 2:** Demographic and clinical characteristics of patients developing APS type 4.

Disease	Number of patients (%)	Sex M/F	Overall mean age of diagnosis (years)	Median latency before second manifestation (years)	Total follow-up from first manifestation (years)
Type I diabetes mellitus	72 (75.0%)	32/40	22.0 ± 15.1	11.5 (1–46)	26.7 ± 12.6
Celiac disease	9 (9.4%)	4/5	24.0 ± 19.8	13 (1-29)	24.2 ± 11.0
Vitiligo	4 (4.2%)	2/2	19.5 ± 15.2	9 (2-20)	24.3 ± 12.3
Psoriasis	3 (3.1%)	2/1	18.3 ± 6.5	9 (6-16)	31.3 ± 21.1
Inflammatory bowel disease	2 (2.1%)	1/1	34.0 ± 26.9	19.5 (19-20)	25.5 ± 0.7
Rheumatoid arthritis	2 (2.1%)	0/2	28.5 ± 3.5	14.5 (7-22)	23.5 ± 9.2
Premature ovarian failure	1 (1.0%)	0/1	30	8	22
Seronegative arthritis	1 (1.0%)	0/1	42	2	13
Ankylosing spondylitis	1 (1.0%)	1/0	42	10	28
Primary biliary cirrhosis	1 (1.0%)	1/0	41	20	32

**Figure 1:**
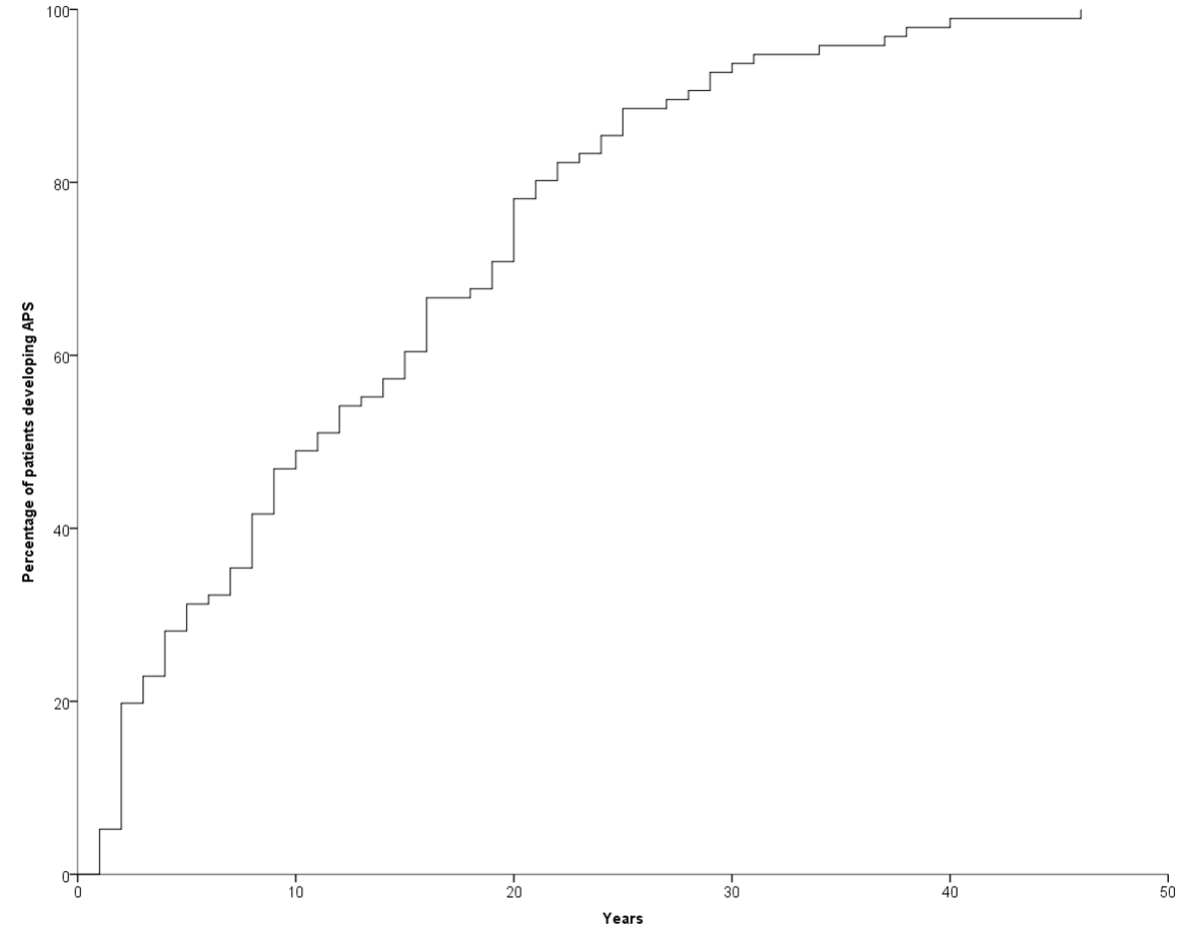
Temporal trend of manifestation of the second disease.

**Figure 2:**
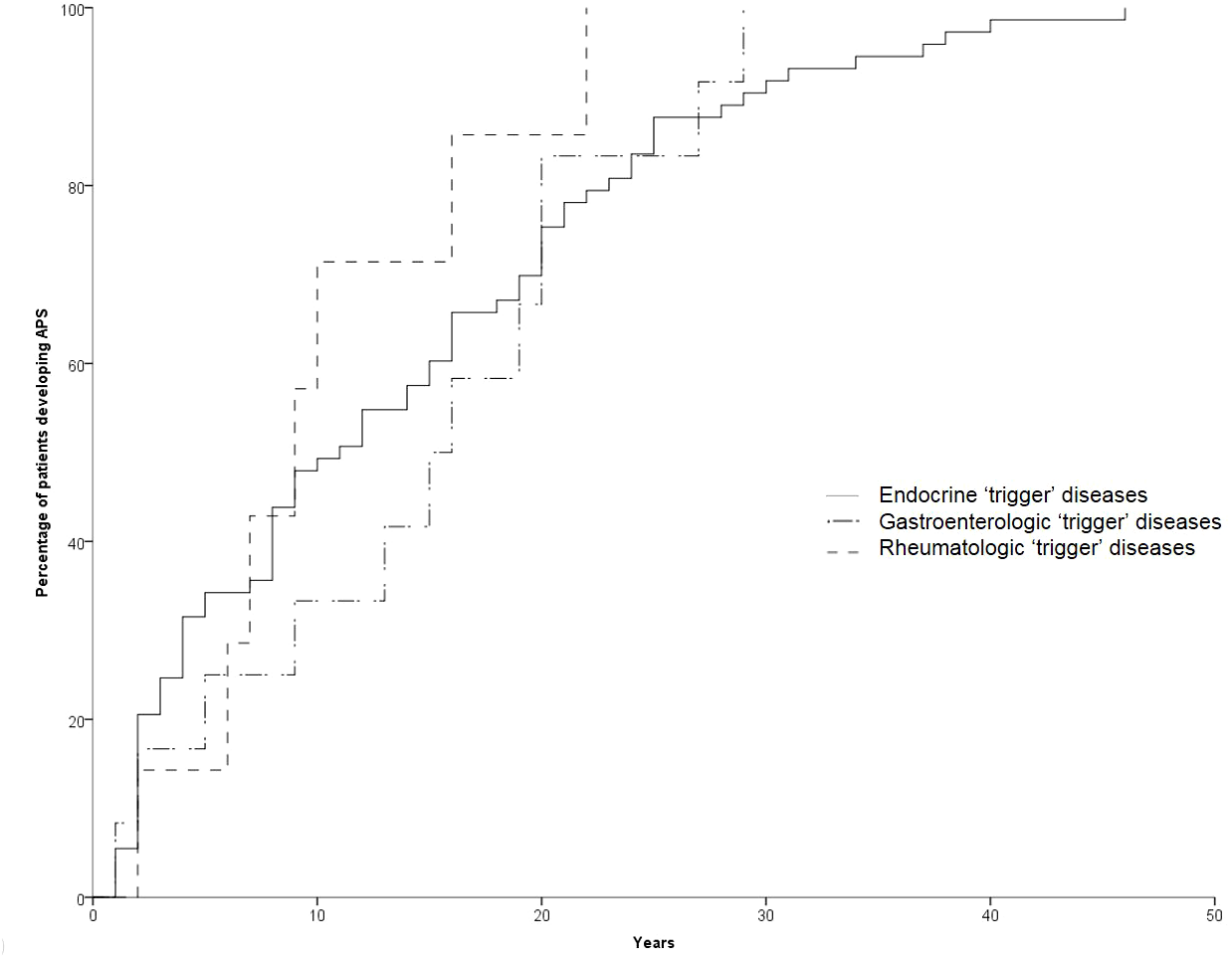
Temporal trend of manifestation of the second disease according to the first “trigger” disease.

**Figure 3:**
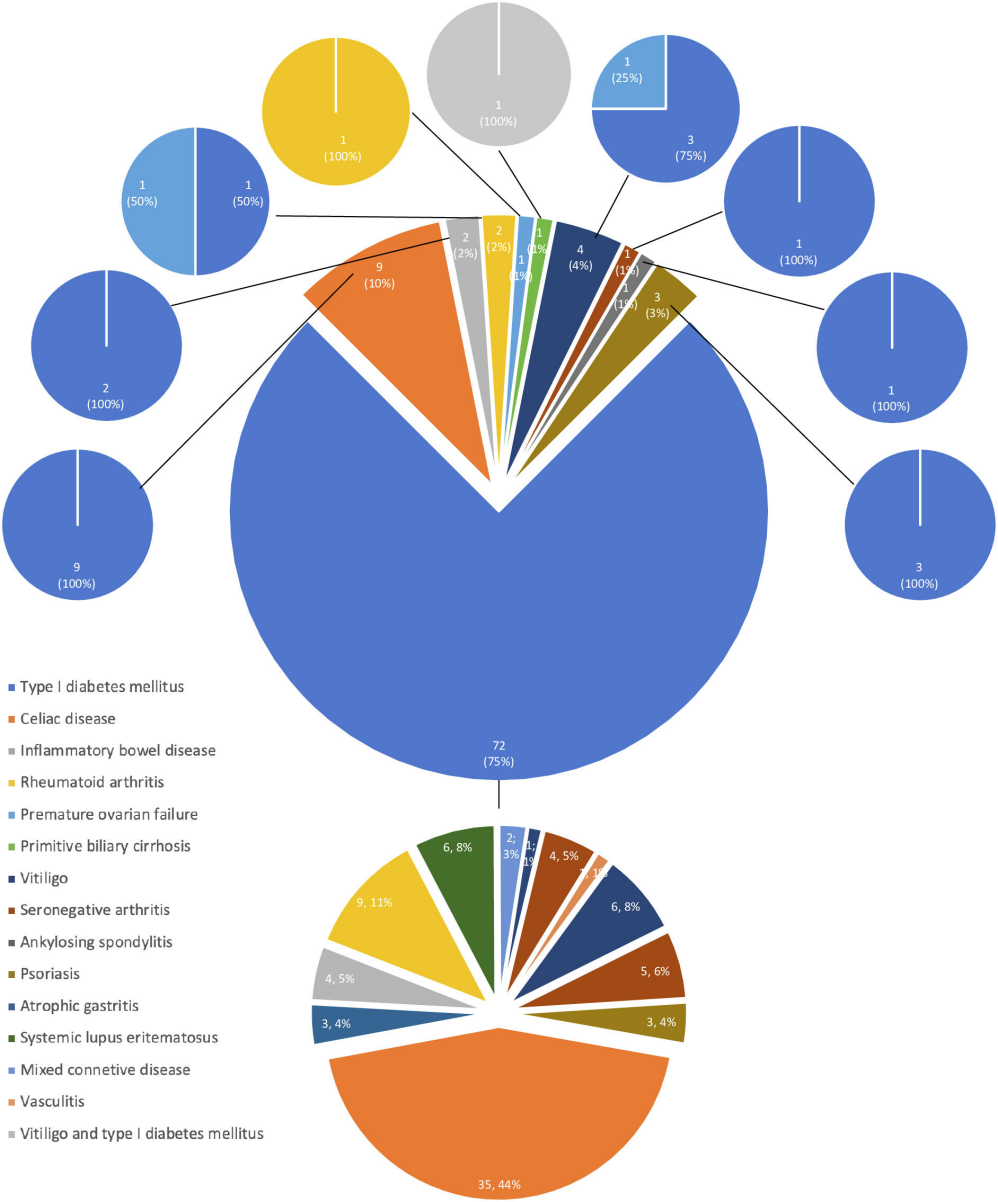
Developing features of APS in accordance with the first disease.

The development of APS over the years (Group 2) is shown in **Figure 1**. The diagnosis was reached after a median latency of 11 years (range 1-46) [10 (range 1-46) vs. 11 (range 1.38), M/F, p=.198]. 50% of subjects developed APS within ten years (**Figure 1**). No difference was found after subdividing the first clinical manifestation into the different diseases to which they referred [11 (range 1-46) vs. 15.5 (range 1-29) vs. 9 (range 2-22) for endocrine, gastroenterological, and rheumatologic diseases, respectively, p=0.643] (**Figure 2**). Five patients developed two subsequent concomitant manifestations (2 subjects had both connective tissue disease and inflammatory bowel disease, 1 vitiligo and T1DM, 1 vitiligo and celiac disease and 1 celiac disease and rheumatoid arthritis); 3/96 (3%) showed a third disease with latency from the onset of the second of 34 years (range 14-36). The developing features of APS are shown in **Figure 3** and **Table 3**. The most frequent pathway was T1DM followed by celiac disease occurring in 35 (48.6%) patients (15 males) with a latency of 4 years (range 1-28); conversely, all the celiac patients developed T1DM with a latency of 14 years (range 1-29) (**Table 3**).

**Table 3:** Pathway of subsequent APS manifestation according to the first clinical feature.

Second disease	Number of patients (%)	Sex M/F	Latency and range (years)	Latency M/F (years)	*p*
Type I diabetes mellitus as first disease					
Celiac disease	35 (48.6%)	15/20	4 (1 – 28)	M 4 (1-28)	.198
				F 4.5 (1-25)	
Rheumatoid arthritis	9 (12.5%)	2/7	20 (3 – 38)	M 19.5 (14-25)	.397
				F 20 (3 – 38)	
Systemic lupus erythematosus	6 (8.3%)	2/4	11 (2 – 37)	M 22.5 (8-37)	.009
				F 9 (2-20)	
Vitiligo	6 (8.3%)	4/2	20 (2 – 40)	M 20 (2-40)	.696
				F 15.5 (9-22)	
Seronegative arthritis	5 (6.9%)	2/3	20 (12 – 36)	M 23 (12-34)	.512
				F 20 (15-36)	
Inflammatory bowel disease	4 (5.6%)	2/2	25 (16 – 34)	M 31.5 (34-29)	.054
				F 18.5 (16-21)	
Multiple sclerosis	4 (5.6%)	1/3	24 (14 – 30)	M 24	NA
				F 24 (14-30)	
Atrophic gastritis	3 (4.2%)	1/2	8 (4 – 16)	M 4	NA
				F 12.0 (8-16)	
Psoriasis	3 (4.2%)	3/0	23 (12 – 46)	M 23 (12-46)	NA
Mixed connective tissue disease	2 (2.8%)	1/1	25 (21 – 29)	M 29	NA
				F 21	
Vasculitis	1 (1.4%)	1/0	8	M 8	NA
Immune thrombocytopenia	1 (1.4%)	1/0	3	M 3	NA
Celiac disease as first disease					
Type I diabetes mellitus	9 (100%)	4/5	13 (1 – 29)	M 15.5 (2-27)	.684
				F 9 (1-29)	
Vitiligo as first disease					
Type I diabetes mellitus	3 (75%)	2/1	7 (2 – 20)	M 11 (2-20)	NA
				F 7	
Premature ovarian failure	1 (25%)	0/1	11	F 11	NA
Psoriasis as first disease					
Type I diabetes mellitus	3 (100%)	2/1	9 (6 – 16)	M 7.5 (6-9)	NA
				F 16	
Inflammatory bowel disease as first disease					
Type I diabetes mellitus	2 (100%)	1/1	19.5 (19 – 20)	M 19	NA
				F 20	
Rheumatoid arthritis as first disease					
Type I diabetes mellitus	1 (50%)	0/1	22	F 22	NA
Premature ovarian failure	1 (50%)	0/1	17	F 17	NA
Premature ovarian failure as first disease					
Rheumatoid arthritis	1 (100%)	0/1	7	F 7	NA
Primary biliary cirrhosis as first disease					
Vitiligo and Type I diabetes mellitus	1 (100%)	1/0	20	M 20	NA
Seronegative arthritis as first disease					
Type I diabetes mellitus	1 (100%)	0/1	2	F 2	NA

NA, not applicable.

The demographic characteristics of all patients affected by autoimmune polyglandular syndrome Type 4 are reported in **Table 4**. In detail, 108/111 (97%) subjects (51males) showed T1DM. In 87/108 (81%) (40 males) it occurred as the first clinical feature of APS with a mean age at the onset of 22.3 ± 14.8 years old. Celiac disease was diagnosed in 57 (51%) patients (27 males), occurring as first disease in 22 (39%) subjects (12 males) at the age of 23.7 ± 16.3 years old. Rheumatoid arthritis was diagnosed in 12 (11%) patients (2 males). It occurred as first disease in two females (28.5 ± 3.5 yrs) followed by premature ovarian failure (POF) or T1DM after 17 and 22 years, respectively. Vitiligo was diagnosed in 11 (10%) patients, and in 4 (2 males) as first manifestation followed by three cases of diabetes mellitus and one case of POF. This last condition was diagnosed in 3/60 women (5%), showing anti-21-hydroxylase antibodies.

**Table 4:** Overall demographic and clinical characteristics of APS patients.

Disease	Number of patients (%)	Sex M/F	Overall mean age of diagnosis (years)	Mean age of diagnosis M/F (years)	*p*	Disease 1^st^ – 2^nd^ – 3^rd^
Type I diabetes mellitus	108 (97.3%)	51/57	25.4 ± 16.4	M 25.2 ± 16.8	.828	87 – 20 – 1
				F 25.6 ± 16.2		
Celiac disease	57 (51.4%)	27/30	25.7 ± 15.3	M 23.6 ± 14.2	.767	22 – 35 – 0
				F 25.7 ± 15.3		
Rheumatoid arthritis	12 (10.8%)	2/10	40.5 ± 15.0	M 36.7 ± 27.4	.032	2 – 10 – 0
				F 41.7 ± 12.0		
Vitiligo	11 (9.9%)	7/4	37.1 ± 18.8	M 37.8 ± 24.8	.009	4 – 7 – 0
				F 36.0 ± 5.4		
Inflammatory bowel diseases	6 (5.4%)	3/3	47.2 ± 19.7	M 45.3 ± 7.5	.068	2 – 4 – 0
				F 49.9 ± 30.0		
Systemic lupus erythematosus	6 (5.4%)	2/4	14.2 ± 13.0	M 22.5 ± 20.5	.018	0 – 6 – 0
				F 10.0 ± 8.5		
Multiple sclerosis	6 (5.4%)	1/5	29.7 ± 8.6	M 42	NA	2 – 3 – 1
				F 27.2 ± 6.8		
Psoriasis	6 (5.4%)	5/1	30.0 ± 18.3	M 33.6 ± 17.9	NA	3 – 3 – 0
				F 12		
Seronegative arthritis	5 (4.5%)	1/4	27.8 ± 17.1	M 12	NA	1 – 3 – 1
				F 31.8 ± 16.9		
Atrophic gastritis	3 (2.7%)	1/2	39.0 ± 19.5	M 59	NA	0 – 3 – 0
				F 20.0 ± 12.7		
Premature ovarian failure	3 (5.0%)	0/3	36.3 ± 5.7	F 36.3 ± 5.7	NA	1 – 2 – 0
Mixed connective tissue disease	2 (1.8%)	1/1	58.5 ± 19.1	M 45	NA	0 – 2 – 0
				F 72		
Immune thrombocytopenia	1 (0.9%)	1/0	17	M 17	NA	0 – 1 – 0
Vasculitis	1 (0.9%)	1/0	63	M 63	NA	0 – 1 – 0
Primary biliary cirrhosis	1 (0.9%)	1/0	41	M 41	NA	1 – 0 – 0
Ankylosing spondylitis	1 (0.9%)	1/0	42	M 42	NA	1 – 0 – 0

NA, not applicable.

## 4 Discussion

The present study describes for the first time the prevalence of APS type 4 among a large series of patients affected by autoimmune diseases.

Autoimmune polyglandular syndromes are rare orphan diseases encompassing a wide spectrum of autoimmune disease, with the involvement of endocrine and non-endocrine organs (1, 10). There are many studies that describe these syndromes (2, 3, 6–8, 11), but few articles, mainly series of case reports, have focused on APS type 4. This can also be due to the fact that some Authors consider APS type 2, 3 and 4 as different phenotypes of the same underlying disease mechanism classifying them as a single entity (2, 7). However, taking in account that each APS type is uniquely characterized by a unique endocrinopathy, we recognized them as separate entity in agreement with the Resource of Rare Disease Co-founded by the Health Programme of European Union (1).

To the best of our knowledge, there is no data on its prevalence reported worldwide (1, 14). One possible explanation could be the large heterogeneity of conditions characterizing the syndrome, which can lead to over- or under-diagnosis. In fact, the few studies about APS type 4 include patients affected by autoimmune thyroid disease, hypoparathyroidism and/or adrenal insufficiency that, according to the Orphanet definition, exclude *a priori* APS type 4 (1). Another reason could be the scarcity of studies performed in a large series of patients affected by “trigger” diseases referred to a single center; we believe that this is the key point of the present study. As reported above, we carefully selected patients affected by APS type 4, in accordance with the ORPHAcode, among those referred to our units for autoimmune diseases. Keeping in mind our data and taking into account the population of our province (1,253,993 inhabitants) (15), the estimated prevalence of APS type 4 is 9 cases per 100,000, thus classifying it as a rare disease as defined by the European Union Regulation on Orphan Medicinal Products (1).

Again, no data about sex distribution are reported. However, Frommer and Kahaly showed a sex ratio (M:F) of 1:3 in adult patients affected by APS type 2, 3 and 4 (11). On the contrary, we found a male to female ratio of 1:1 in our sample. Although most autoimmune diseases are more common in females, no sex difference in the overall incidence of youth T1DM is demonstrated (16, 17). Our ratio appears to be in keeping with literature, since T1DM is present in almost all patients (97%). T1DM is a key element *in* and *for* the diagnosis of APS type 4. In fact, T1DM was the leading indicator of APS type 4 in 87/111 (78%) subjects and in 21/111 (19%) permitted the diagnosis as the second manifestation of the syndrome.

About ten years ago, Van den Driessche et al. suggested a flowchart for screening and follow-up of few associated autoimmune disorders (autoimmune thyroid diseases, atrophic gastritis, celiac disease, Addison’s disease, and vitiligo) in patients affected by T1DM. With the exception of thyroid diseases, the authors proposed an annual screening for the first three years and then once every 5 years; the thyroid should be checked annually. However, this recommendation covered all the autoimmune syndromes except APS type 1 (18). Our data, showing that 50% of patients developed APS type 4 within the first ten years, don’t suggest any particular follow-up time and, more importantly, don’t specify any particular disease (**Figure 1**). In other words, these data suggest a lifelong follow-up, although the cost-effectiveness of this is yet to be proven. However, in 68% of patients with T1DM developing celiac disease this occurred within 10 years. For this reason, the screening of celiac disease should be done very early since therapy of T1DM is very difficult in patients with unknown celiac disease (19). In addition, it is important to emphasize that 5% of women in our series developed POF. These data reinforce what Li et al. previously reported on the prevalence of autoimmune disorders in women affected by POF (20). As is well known, this condition severely affects women’s lives (21–25). For this reason, we suggest and recommend that gynecologists perform regular check-ups with complete blood exams during childbearing age for these patients.

Celiac disease was the second most frequent disease among our patients (57/111, 51%). Literature data show that females are affected approximately twice as often as males, although the ratio varies depending on the strategy used to identify cases (26). Our male to female ratio was instead 1:1 among our patients. As reported above, this could be because all these patients were also affected by T1DM, and celiac disease is known to be closely associated with type 1 diabetes mellitus (27–31). These conditions share the same HLA susceptibility alleles, specifically DR3/DQ2 and DR4/DQ8 molecules (32). However, the co-occurrence of the disorders is not fully explained by shared genetic risk loci (30). Few studies in animal models (33, 34) and in humans (35) have shown that celiac disease may trigger autoimmune processes leading to diabetes. On the other hand, some authors have reported the development of celiac autoantibodies after the onset of diabetes (30, 36, 37). In line with this finding, 61% of our patients developed celiac disease 1-28 years after T1DM diagnosis (**Table 3**).

As is widely known, it is the pathologic response to self- or autoantigens that characterize autoimmune diseases. It is generically categorized as autoimmunity or autoreactivity, which covers a wide range of clinical disorders (38). The pathogenesis of autoimmune disease is still largely unknown: familial or genetic, infectious, immunologic, and psychological factors have all been implicated as triggers (39, 40). Consequently, it is reasonable to believe that once the trigger, if any, activates the process, then it can be amplified. Moreover, a recent observational study by Bechi Genzano et al. showed that the circulating immune profile was similar in patients diagnosed with T1DM and those affected by other autoimmune diseases. The authors demonstrated an increase in CD4 T-cells and a consensual reduction in natural killer (NK) cells and CD8 T-cells, underlying a similar pathogenetic pathway (31). In addition, major autoimmune disorders share much of their molecular background, including class II HLA haplotypes (41–45). Houcken et al. demonstrated that protein tyrosine phosphatase non-receptor type 22 (*PTPN22*) and cytotoxic T-lymphocyte associated protein 4 (*CTLA-4*) polymorphisms are also associated with autoimmune polyglandular syndromes (46). Most recently, evidence by Fichna et al. appears to show that BTB domain and CNC homolog 2 (*BACH2*) gene polymorphism, implicated in lymphocyte differentiation and function, may also promote multitarget autoimmunity (47). Genetic screening is growing popular to identify patients at risk of autoimmune disorders, though this remains too expensive to be included in routine clinical management and is not readily available. Furthermore, there is still a lack of evidence as to the usefulness of this process in daily clinical practice. Therefore, we have no genetic screening data for the patients in our study. It is also widely accepted that autoantibodies play a crucial role in the diagnosis of autoimmune disease, especially in the early phases when the patient is still asymptomatic and biochemical markers are normal (7). In a minority of cases, however, patients may not show any autoantibodies, a condition that is referred to as seronegative autoimmune diseases, as recently reviewed by Lenti et al. (48). In these cases, the diagnosis is more challenging and must rely on clinical features and other available tests, often including histopathological evaluation and radiological diagnostic tests. In all our patients, the diagnosis was confirmed by the presence of serum autoantibodies and/or histopathological specimens.

The main limitations of the present study are its retrospective nature and the possible patient drop-out during follow-up after the diagnosis of the first “trigger” disease. The latter is a key point, as it could reduce the prevalence of APS in our cohort of patients. Unfortunately, we have no data on patient drop-out precisely due to the retrospective nature of the study. Indeed, it is unlikely that all these patients were affected by APS type 4. Finally, we must state that a possible bias of case selection is possible since the data collection has been performed in a reference center for autoimmune diseases. However, the large set of patients, the careful selection procedure and detailed analysis of patient clinical records strengthen our results.

In conclusion, the prevalence of APS type 4 is 9:100,000 people. Type 1 diabetes mellitus, for its high prevalence among our patients, could be the clinical “driver” of this syndrome: for this reason, diabetologists should pay particular attention during clinical examinations of T1DM patients. Our data don’t suggest any particular follow-up time and, more importantly, don’t specify any particular disease, but only indicate a lifelong follow-up. Finally, we recommend regular gynecological evaluations with complete blood exams during childbearing age due to the non-negligible risk of developing premature ovarian failure.

## Data availability statement

The original contributions presented in the study are included in the article/**Supplementary Material**. Further inquiries can be directed to the corresponding author.

## Ethics statement

The studies involving humans were approved by Brescia Ethics Committee (no 5517). The studies were conducted in accordance with the local legislation and institutional requirements. The participants provided their written informed consent to participate in this study.

## Author contributions

Conceptualization and Methodology: CC, CR, FF, and AG. Data curation: EliG VA, EC, VM, IZ, EP, and GG. Formal analysis: EliG and CC. Investigation: All the authors. Project administration: CC. Supervision, validation, and visualization: IP and LaP. Writing - original manuscript: EliG and CC. Writing – review and editing: CC, CR, FF, VA, IZ, EP, GG, EC, and AG. CC, CR, FF, and AG had full access to and verified all the study data and were responsible for the decision to submit for publication. All authors contributed to the article and approved the submitted version. All authors affirm the accuracy and completeness of the data and attest to the fidelity of the study to the protocol.
